# Understanding the implementation, impact and sustainability of Bristol’s unhealthy commodity advertising restrictions policy: a qualitative systems-based study of stakeholder perspectives

**DOI:** 10.1136/bmjph-2025-004102

**Published:** 2026-06-29

**Authors:** Sarah K Harding, James Nobles, Jeremy Horwood, Rowan Brockman, Genevieve Buckland, Zoi Toumpakari, Agnes Nairn, Sarah Blake, Steven Cummins, Russell Jago, Carlos Sillero Rejon, Frank de Vocht

**Affiliations:** 1 Applied Research Collaboration West (NIHR ARC West), University of Bristol, Bristol, UK; 2 Population Health Sciences, University of Bristol, Bristol, UK; 3 School of Health, Leeds Beckett University, Leeds, UK; 4 Centre for Public Health, Bristol Medical School, University of Bristol, Bristol, UK; 5 Centre for Exercise, Nutrition and Health Sciences, University of Bristol, Bristol, UK; 6 Bristol Hub for Gambling Harms Research, University of Bristol, Bristol, UK; 7 Centre for Research in Health and Social Care, University of Bristol, Bristol, UK; 8 Population Health Innovation Lab, Department of Public Health, London School of Hygiene & Tropical Medicine, London, UK

**Keywords:** Public Health, Obesity, Qualitative Research

## Abstract

**Introduction:**

In 2021, Bristol became the first English local authority (LA) outside London to introduce restrictions on advertisements for high fat, salt and sugar products and other unhealthy commodities (alcohol, gambling and payday loans) on LA outdoor advertising sites (bus shelters and some billboards). This study aimed to explore the policy’s acceptability, implementation barriers and facilitators, perceived impacts and considerations for sustaining and wider roll out of the policy.

**Methods:**

22 online semi-structured interviews were conducted with key stakeholders from Bristol-City-Council (n=12), advertising industries (n=3), third-sector advertising and sustainability organisations (n=5) and the Office for Health Improvement and Disparities (n=2). Interview transcripts were analysed using inductive and deductive thematic analyses.

**Results:**

Stakeholders acknowledged the importance of this policy as part of a system-wide approach to decrease consumption and use of unhealthy commodities. However, several barriers to behaviour change were recognised: (1) limited scope for change of outdoor advertising exposure, (2) displacment of adverts for unhealthy comodities and (3) adaptations to advertising copy. To sustain the policy, stakeholders highlighted the need for a formal auditing process and continued reviewing and improving of the policy in response to industry adaptations as well as expanding the policy to include private advertising spaces.

**Conclusion:**

While the policy was seen as a valuable step towards reducing use and consumption of unhealthy commodities, its impact on behaviour may be limited due to the LA owning relatively little outdoor advertising space and by industry adaptations to enable advertising of unhealthy commodities to continue.

**WHAT IS ALREADY KNOWN ON THIS TOPIC**

- Advertising is consistently associated with increased use and consumption of unhealthy commodities. As a result, a growing number of local authorities (LAs) in the UK are introducing or considering policies to restrict the advertising of unhealthy commodities on LA-owned premises. However, little is known about the implementation process of these policies and unintended consequences of such policies.

**WHAT THIS STUDY ADDS**

- This research suggests that policies restricting unhealthy commodity advertising are an important part of a whole systems approach to improving public health. However, the effectiveness of such policies may be limited by the low percentage of outdoor advertising spaces owned by LAs and by the rapid adaptations made by well-funded businesses selling unhealthy commodities to overcome reduced purchasing.

**HOW THIS STUDY MIGHT AFFECT RESEARCH, PRACTICE OR POLICY**

- Policies such as this could be strengthened by incorporating an internal monitoring process to ensure adherence to the policy. It would also benefit from continued reviewing and adaptation to keep up with evolving industry practices and by seeking to apply the policy to additional outdoor advertising sites.

## Introduction

 Non-communicable diseases such as obesity, type II diabetes and cardiovascular disease are omnipresent in high-income countries, and more recently, in low- and middle-income countries.^1–3^ The forces driving these global health challenges are complex, interconnected and share many commonalities. The commercial determinants of health, characterised as the strategies and approaches used by the private sector to promote products and influence choice in ways detrimental to health,^4^ are an integral part of this puzzle. Within this sits the advertising and marketing of unhealthy commodities.

The evidence base consistently demonstrates a link between food marketing and the purchasing and consumption of unhealthy products.^5–7^ Advertising and marketing can play a role in widening health inequalities, with evidence suggesting that individuals from deprived backgrounds or from ethnic minority groups are disproportionately targeted with unhealthy food and drinks advertisements.^8–10^ Children and adolescents are often specifically targeted due to their exploitable cognitive vulnerabilities, purchasing power and the potential for long-term brand loyalty.^11–13^

In England, outdoor advertising (eg, on advertising billboards and on bus stops) is estimated to reach over 98% of the population each week.^14^ A quarter of these adverts promote food and drink products,^9,15^ most of which are classified as unhealthy.^9^ Local and national governments hold legislative powers to regulate how products are marketed to their populations.^16^ A growing number of local authorities (LAs) in England are introducing or considering policies restricting advertising of unhealthy commodities on LA-owned premises.^17^ London was the first to legislate against advertising food and non-alcoholic drinks which are high in fat, salt and/or sugar (HFSS) across the entire Transport for London (TfL) network. The policy was associated with a reduction in average weekly household energy purchased from HFSS products per household of 1001.0 kcal (95% CI 456.0 to 1546.0).^18^

Bristol-City-Council was the first LA outside of London to introduce an unhealthy commodities advertising restriction policy. Bristol is a large city in England with a population of 472 500^19^ within the city limits (and a larger population in the wider city region). When referring to Bristol we are referring to the area served by Bristol-City-Council, other areas within the city are served by a different LA. The Bristol Advertising and Sponsorship Policy (herein referred to as ‘the policy’), introduced in November 2021 and implemented from April/May 2022, restricts the advertising of HFSS products, alcoholic drinks, tobacco or tobacco substitutes, gambling and loan advances. The policy applies only to LA-owned outdoor advertising sites and not commercially owned sites. LA-owned sites comprise approximately 30% of total advertising sites (according to Bristol City Council^20^).

The Bristol Evaluation and Advertising Restrictions (BEAR) study aimed to evaluate the implementation and impact of this advertising policy through a mixed-methods, natural experiment research design, exploring impact on exposure, purchasing and consumption. Our quantitative work found that objectively measured advertising exposure on LA-owned Bristol bus shelters decreased from 11% to 0.8% at 18-month follow-up. There was no measurable impact of the policy on consumption/use of unhealthy commodities or purchasing of HFSS products.^21,22^ A core part of the BEAR study, and the aims of this paper, was to take a systems perspective to understand the: (1) policy implementation processes and associated barriers and facilitators; (2) perceived impact of the policy and (3) recommendations for sustainability and wider policy roll out.

## Materials and methods

### Study design

We undertook qualitative research with key stakeholders involved in, or affected by, the policy. This research builds on our previous work which explored the barriers and facilitators at the policy development stage.^20^ Ethical approval was obtained from the University of Bristol Faculty of Health Sciences Research Ethics Committee (9754). A completed Consolidated criteria for Reporting Qualitative studies checklist^23^ is provided in online supplemental file 1.

### Recruitment strategy

Purposive sampling was initially used to recruit stakeholders either directly involved in the policy development/implementation or who were affected by its enactment. This includes those from the LA involved in designing and leading the policy (Public Health, Strategy and Digital LA communications team), and those in the LA affected by the policy (namely Public Transport and Economic Development), as well as local councillors/politicians. Third-sector advertising and sustainability organisations involved in policy design and implementation, as well as stakeholders working for outdoor advertising or foods and drinks companies, were also invited to interview. We specifically targeted companies who had advertised on Bristol-City-Council-owned advertising spaces before and after the policy implementation, as identified in the wider BEAR study. Those involved in the pre-implementation study^20^ were invited to participate again. Snowball sampling was used to identify further stakeholders. Potential participants were invited to participate via email and were provided with a participant information sheet detailing the purpose of the research, the research team and what participation involved.

### Data collection

Semi-structured interviews were conducted between January and October 2024. The interview topic guide was developed within the research team and through stakeholder discussion. Interviews explored: policy implementation, barriers and facilitators to implementation, its acceptability, how it aligns with the wider policy context, its perceived impacts and considerations for other LAs. Specific interview questions were tailored to each participant’s expertise and knowledge. All interviews were carried out before the quantitative findings were generated, and as such, interviewees were not aware of the policy’s effectiveness as determined via the BEAR study. One-to-one interviews were carried out online by SKH (using MS Teams or Zoom), audio recorded and transcribed verbatim and then checked for accuracy. Interviews ranged from 25 to 60 min. Each participant was interviewed once. Written or verbal (recorded) informed consent was gathered prior to interview. Throughout the data collection and analysis process, SKH kept reflexive notes, impressions of the interview and thoughts about the data collection and analysis. These informed the thematic analysis. Data were collected until data saturation was reached, and this was determined by discussions among the research team involved in data analysis (SKH, JN and RB).^24^

### Data analysis

Data were analysed thematically^25^ in NVivo (V.14). All transcripts were read by SKH for data familiarisation. Initial codes and coding framework were developed inductively by the lead researcher (SKH) and four interview transcripts were second coded by JN and RB to refine the coding framework. The themes were generated based on the codes and were reviewed for coherence in the later stages of the analysis while also reflecting the content of the transcripts. Any disagreements or uncertainties related to coding were resolved through discussion between SKH, JN and RB. Remaining transcripts were coded by SKH.

## Results

22 participants were interviewed (see table 1). An additional two employees from third-sector and one from Bristol-City-Council were approached and declined an interview and 23 food and/or drinks companies and one other advertising company were approached and either declined an interview (n=2) or did not respond (n=22). Our analysis led to four major themes: (1) policy impacts, (2) facilitators and barriers of policy implementation, (3) barriers to behaviour change (or impact) and (4) sustaining and further improving the policy. Subthemes are presented as subsections under each major theme.

**Table 1:** Participant information

Participants/stakeholder type (n=22)	Department/organisation
Local authority (n=12)	Public health
	Communications
	Economics
	Public transport
	Policy strategy
	Elected councillors
Third sector (n=5)	Ad block and adfree cities
	Food active
	Sustain
Industry (n=3)	Gambling industry
	Advertising company
Office for Health Improvement and Disparities (n=2)	Omitted for anonymity

### Policy impacts

We noted several pertinent impacts perceived to be associated with policy implementation, these were: pressure for further change (both within and outside of the LA), improved reputation of the LA and its contribution to a whole systems approach to promote public health.

#### Increased pressure for further change

Third-sector and LA participants often stated that this policy increased pressure on policy makers for further change within the LA by acting as a springboard for further conversations and change within the LA. For example, it could be used as a template for restrictions of other products such as high-carbon products. Additionally, some also felt that it increased the pressure for the LA to influence advertising through other means. For example, the planning policy took a stronger stance on new digital advertising boards, and the LA has rejected planning applications for these which could reduce the overall volume of advertising of unhealthy commodities.

Being able to reference [the policy] in objections to further applications for more screens as well. So being able to reference back to the policy and say, ‘Well you said that this content isn’t appropriate, so we don’t wanna see more screens showing more content.’ (Third-sector participant)

There was a strong sense among third-sector and LA participants that the implementation in Bristol encouraged other LAs to consider, or adopt, similar policies. The policy team had presented the policy at national events and had conversations with other LAs. Bristol-City-Council had also been used as an exemplar by third-sector advertising and sustainability organisations. Interviewees stated that the policy implementation in Bristol had set a precedent for other LAs, enabling them to feel confident and safe about adopting their own. Interviewees noted that there has been a historic fear of lawsuits regarding advertising restrictions but this has reduced as more LAs have begun developing such policies.

You get confidence that the world does not end if you put this in place, that your entire structure of a council doesn’t become bankrupt, that you can do something to support the environmental impact of poor advertising on people’s health…just knowing that other places are doing it gives confidence to the leadership of local authorities (OHID participant)

Additionally, some participants stated that LAs, such as Bristol-City-Council, having an advertising restrictions policy may encourage national level change around advertising restrictions.

#### Improved reputation of the LA

Many participants from the LA and third-sector organisations expressed that the policy improved the reputation of the LA and associated trust from the public.

Where I’ve probably seen more communication about it and more impact has been really from a reputational point of view. So I’ve seen sort of more correspondence and had more comments about the principle that we’ve got this policy and that that’s positive as opposed to sort of the direct outcomes of it. (LA participant)

Other participants, primarily those from industry, expressed that policies such as this one are merely about improving reputation as opposed to taking more meaningful action which have a direct impact on health behaviours.

#### Contribution to the whole systems approach to promote public health

Several third-sector, LA and Office for Health Improvement and Disparities (OHID) participants said that this policy contributed to a whole systems approach to promote public health. While on its own, the policy might not have a measurable impact, interviewees stated that the policy sits alongside many others which collectively are perceived to re-shape the obesogenic environment in Bristol.

#### Impact on revenue

The main revenue stream for the LA, which this policy may affect, is the bus shelter advertising contract which is managed by the public transport team. LA participants reported that this policy had minimal impact on income because the advertising spaces were still being filled by advertisements for alternative products.

since the policy’s been in place, we’ve procured the bus shelter contract and we actually saw an increase in the revenue there…. So we absolutely didn’t see, when we tested the market, this having an impact in terms of reducing income or ability to advertise. (LA participant)

Contrary to this, industry participants or participants whose figures were provided to them by industry reported that this policy had a negative impact on income for the LA

since getting rid of HFSS, has there been a decline in revenue yes, because there’s no HFSS advertising and six out of the top 10 advertisers at one point in ((Bristol)) fitted into that category, so it’s quite a shift. (Industry participant)

Participants from both industry and LA noted that there is difficulty in understanding the true changes to revenue as (a) since the implementation of the policy, the contract between LA and the advertising company has changed, altering the balance between fixed payments and profit-share and (b) there are unknown opportunity costs as we do not know what the income from the advertising contracts would have been if policy had not been in place. Some participants within the LA acknowledged that if there were a revenue loss from advertising, they viewed it as a short-term loss for long-term gain regarding costs of poor health.

That’s the big question we always get asked, is ‘has it lost you money?’ My instinct would be yes it probably has a little bit but if you’re talking long-term the health costs that might have been gained then I think that far outweighs the potential loss. (LA participant)

### Facilitators of implementation

Stakeholder perceptions were mixed regarding the ease of policy implementation. Senior LA employees described the implementation as straightforward whereas those involved in the daily operations noted substantial workload implications. We identified several key factors which supported policy implementation: (1) housing the policy in communications—rather than public health—department, (2) resources and training within the LA and (3) having a clear definition for product restrictions.

#### Housed in the LA communications team

Participants noted that having the LA’s communications team lead the policy implementation was beneficial due to their expertise and understanding of how local advertising works. Many observed that having a dedicated person responsible for the policy helped these processes and that the communications team was the most logical place for centralising this policy.

I think it does help having it embedded into the comms team, because it’s their bread and butter, they have to do it. (LA participant)

#### Resources, training and communications within the LA

Some participants stated that training and resources within the LA supported policy implementation. Resources were made available on the staff intranet and included worked examples demonstrating how to apply the nutrient profile model (NPM) to assess whether products met the policy criteria. They were developed with support from a third-sector organisation. Training and communication were informal and took place in meetings and via emails, making relevant teams aware of the policy details and how it may affect their role.

Training, people getting used to the whole process. So where we were getting these edge cases we’d sort of capture that example and use it in team briefings or use in training for new staff to help people understand. (LA participant)

#### Clear definition of HFSS foods and drinks

Participants involved in the implementation of the policy agreed that using the nutrition profile model to classify a food or drink as a HFSS project eased the implementation. They stated that its objectivity reduced ambiguity around this aspect of the policy.

They understand, because it is based on this nutritional profile model that’s definitive. It’s not subjective. A product either complies or it doesn’t. I think it’s fairly black and white. (LA participant)

### Barriers to implementation

Several key barriers to policy implementation were also identified: (1) fragmented advertising sites and contracts across the LA, (2) lack of information sharing and (3) competing priorities between-teams and within-teams at the LA.

#### Fragmented advertising sites and contracts across the LA

Participants from the LA and third-sector noted that advertising and sponsorship contracts within the LA are distributed across different LA departments (ie, not all the advertising and sponsorship contracts are held in the same department). Participants raised this as a barrier to implementation as it makes it difficult to identify which contracts needed to be modified after the policy was introduced. Moreover, the advertising estate (particularly in cities) is fluid and as such keeping an up-to-date list of the estate and therefore advertising contracts is difficult.

different types of billboards, different departments in councils have had different contracts with different providers for their different advertising space. It was messy in the extreme. (OHID participant)

#### Lack of information sharing

There was a lack of willingness and/or capability (ie, commercial sensitivity) across LA teams to share information regarding advertising sites and contracts, data and financial implications of this policy. This was primarily due to concerns of LA teams believing that this information is sensitive and can not be shared beyond the team holding the contract. Lack of information sharing is also apparent between advertising companies and the LA, with the advertising companies holding the advertising data and not sharing it with the LA. This lack of information sharing makes implementing and sustaining the policy more difficult and has implications for accurately reviewing the policy, for example.

There’s also often a real difficulty with getting hold of advertising contracts by teams that don’t hold advertising contracts. So Public Health trying to get hold of the latest advertising contracts across the council. (Third-sector participant)

#### Competing priorities

The policy had caused friction between LA departments following its implementation due to competing priorities within the LA. Some LA participants, particularly those concerned with income generation, believed the policy clashed with their objectives and that it had been solely developed from a public health perspective. Those responsible for high street regeneration, promotion of LA-owned markets or night-time economy found the policy too restrictive, working against their own targets.

You’d think that they’re all one council trying to work towards the same aim, but understandably Public Health aims are actually vastly different from commercial teams aims. (Third-sector participant)

Capacity and capability limitations within teams was also raised as an implementation barrier. Participants from the LA noted that time constraints meant they could not devote sufficient time to support the implementation of this policy due to other priorities. For example, there were plans to create a panel to more formally discuss any adverts suspected to be non-compliant as opposed to the current ad-hoc approach, but these did not materialise due to capacity limitations. This limited capacity also has had an impact on the reviewing and policing of the policy.

### Barriers to behaviour change (or impact)

Participants from LA, industry and the third-sector alluded to reasons why the policy may have limited impact on the use and consumption of unhealthy commodities.

#### Limited scope for change of outdoor advertising exposure

This policy only applies to LA-owned spaces in the city mainly bus shelters and some billboards but does not apply to privately owned advertising spaces. So only a small portion of advertising in the city are affected by this policy. Many participants supported the notion that this limits the policy’s effectiveness as the public in Bristol are still exposed to advertising for unhealthy commodities on privately owned spaces.

I think probably the biggest disappointment for people is that they still do see a lot of junk food advertising because the policy only covers a fraction of the billboards in Bristol. (Third-sector participant)

There is also limited scope for change within the LA-owned spaces as some complimentary advertising restrictions were already in place. In the contract between the LA and outdoor advertising company who manage the bus shelter advertisements there already were restrictions on advertising on alcohol, payday loans and gambling prior to the introduction of this policy. In addition, the outdoor advertising company which manages the advertising on Bristol bus shelters have their own policy which restricts advertising of HFSS products within 200 m of schools, which they claimed applies to around 30% of the LA-owned advertising space.

Third-sector and LA participants raised that contract lengths between advertisers and the LA, from 10 to 25 years, was problematic for imposing the policy. For example, in Bristol the policy does not yet apply to the LA-owned digital pavement billboards as the contract pre-dates the policy. Third-sector participants stated how other LAs in England had tried to implement break clauses (changes applied to contract terms within contract), though none had been successfully negotiated to date. It was not clear whether and how the policy could be applied to existing contracts in LAs.

#### Displacement of adverts for unhealthy commodities

The impact of this policy may also be limited due to industry transferring advertising to other spaces and platforms and there being a ubiquity of advertising spaces for this. Industry participants stated that if a product could no longer be advertised on LA-owned spaces it would instead be advertised on the privately owned billboards in the city. They may also be displaced to LA-owned advertising spaces in surrounding regions which do not have this policy. Additionally, participants noted that the advertising may also shift from outdoor to online and this may be achieved via targeted advertising based on location. Such industry adaptations are made to ensure the advertisements still reach their target audience.

if you were to take the physical kind of advertising out of public spaces, of course, then they'd still have digital…they'd still have access to people’s phones, and…they would be able to have the resources to ensure that if you're walking through Bristol city centre then they'd be able to pay for targeted adds to appear on someone’s Twitter feed, for example, because they know they can't advertise in Bristol city centre… So, they have very sophisticated ways around it (Industry participant)

#### Adaptations to advertising copy

Participants from industry, the LA and third-sector organisations gave examples of where industry have adapted their advertising copy to enable advertising to continue. For example, attempting to use the same branding of the full sugar drink version of a drink but labelling the product as sugar free on the advertising copy. There were also examples from LA participants where advertisements for perceived unhealthy products which they assumed non-compliant were compliant on obtaining an NPM certificate, suggesting industry have found ways in which they can continue to advertise less healthy products while still adhering to the policy. Industry participants also highlighted that industry dedicate resources to finding ways in which they can advertise their products under this policy.

People have to constantly innovate and kind of adapt to the regulations in which they're allowed to work in order to kind of protect that bottom line…they have very powerful teams which are dedicated to ensuring that they are working within the guidelines and finding new solutions…it’s just making sure that their advertising practices are carefully designed. (Industry participant)

Currently the policy restricts products and not brands. Brands can still advertise their sugar-free or fat-free versions of a product. Some industry and third-sector participants raised the issue that because some branding is so synonymous with HFSS products that when its non-HFSS products are advertised, brand recognition is triggered in customers, resulting in the purchase of a product from that brand’s portfolio which could be a HFSS product.

### The future of the policy

Factors perceived to be able to sustain and enhance the policy included: continued reviewing and knowledge sharing, improved monitoring of adherence to the policy, restricting additional products and extending to additional advertising spaces.

#### Continued monitoring, reviewing and knowledge sharing

Participants stated that the policy needs to be regularly reviewed and monitored to ensure its success. Continued reviewing would allow for the policy to be strengthened in response to industry adaptations and the ever-changing advertising landscape. Continuous reviewing allows space for the catalytic conversations that advance thinking and action in this space. Continuous reviewing could also be informed by knowledge sharing between LAs. Collaborating with other LAs shares the time and capacity burden for strengthening these policies and LAs can also learn from each other about how best the policy can be strengthened, making them all more robust and less susceptible to industry loopholes.

Companies have since then responded to the policy. They’ve found loopholes. They’ve done all sorts of different things that we are increasingly making those policies stronger as we bring in more policies. Different local authorities bringing in policy and strengthening that wording and also learning more about what good practice looks like. (Third-sector participant)

#### Improved monitoring of adherence to the policy

Most LA advertising space is bus shelters and billboards. This space is managed by third-party providers such as outdoor advertising companies, and it is these providers who are responsible for policy adherence, so it is self-policed by the outdoor advertising industry. There is no formal monitoring process within the LA for checking adherence to the policy and ensuring that the adverts on the bus-shelters and billboards comply. However, there is some informal and ad-hoc monitoring within the LA. Street team LA employees (street cleaners, bus-stop inspectors, etc) are asked to check for non-compliant adverts, and councillors involved in this policy check adverts informally. These employees may not have the expertise to know if an advertisement is compliant, but they can flag suspected non-compliant adverts. There is also external reporting of policy breaches from a local anti-advertising campaign group. Participants noted that most of the reporting of breaches come from this group. Participants acknowledged that there is a need for a formal auditing process within the LA to ensure adherence.

Obviously, it’s really important that it is monitored, and it’s evaluated properly and continually in terms of upholding the quality standards and ensuring that it continues to be implemented. (OHID participant)

If an advert is non-compliant with the policy, it needs to be removed and participants from both third-sector and LA acknowledged responsive corrections by industry in these cases. While one participant informed us that there is a financial consequence if there are several non-compliant adverts, others were unaware of this and believed there is no consequence for non-compliance to the policy.

#### Restrict additional products

Many participants agreed that this policy could be extended in the future by restricting the advertising of additional products such as restrictions on high-carbon products or activities such as aeroplane flights and sports utility vehicles. Participants from the LA and third-sector organisations noted the dichotomy of having many policies which aim to limit the use of high-carbon products (such as the Clean Air Zone and One City Plan) while also allowing for advertising of high-carbon products on LA-owned space.

it’s astonishing that we’ve got on the one hand a load of policies that say we’re trying to reduce car use…yet, at the same time, on our own advertising, particularly on bus shelters, ironically, we’ve got car adverts. So, it just seems a bit of a push me pull you situation. (LA participant)

Participants noted that one of the reasons for this not being included in the current policy was that there was no agreed definition of what encompasses a high-carbon product and that it has not been included in an advertising restrictions policy prior to Bristol’s. However, participants from third-sector organisations raised that such restrictions have since been included in similar policies at other LAs and that there are also legal opinion pieces confirming that advertising for high-carbon products can be restricted by LAs. Some participants from the LA also noted that future versions of the policy could include restrictions on advertising breastfeeding replacement products.

#### Extending the policy to additional advertising spaces

There was a desire among some participants to extend the restrictions to privately owned advertising spaces in Bristol. There was uncertainty around if this was possible within the LA. However, participants suggested some ways in which it could cover more of the outdoor advertising spaces. Participants raised that the LA have existing partnerships in the city (eg, leisure centres, sport grounds and transport companies who manage the trains and buses), and that encouraging these to adopt the policy could increase the advertising estate that this policy covers.

There may be initiatives across the council, which they get local businesses to sign up to so they will already hold some sort of relationship with a bunch of local businesses, which may also be able to then have some influence over a vast amount of advertising estate. So if you think of housing estates, leisure centres actually together they hold quite a lot of – and actually NHS you know a lot of their advertising estates as well. (Third-sector participant)

Additionally, some participants raised concerns about residents of Bristol still being exposed to advertising of unhealthy commodities from advertising spaces owned by surrounding LAs. This is particularly problematic in Bristol where LA boundaries are within the city. Participants suggested that encouraging surrounding LAs to adopt an advertising restrictions policy could help to reduce exposure to unhealthy commodities by Bristol residents.

## Discussion

### Summary of main findings

This research explored stakeholder perceptions of the implementation and impact of the Bristol Advertising and Sponsorship policy. Overall, stakeholders believed this policy increased pressure for further change, that it improved the LA’s reputation and that it was an important aspect of a whole systems approach to support public health. The impact on revenue was unclear; participants from the LA believed it had minimal impact on revenue whereas those from industry expressed that a restrictive policy had a negative impact. Policy implementation was supported by being led by the LA’s communications team, having adequate LA resources available and a clear definition of HFSS products. That said, having fragmented advertising sites and contracts, limited information sharing both within the LA, and between LA and advertising industry, made implementation challenging. Competing priorities between LA departments, primarily between public health teams and income generating teams, also hindered implementation.

Our quantitative analysis of HFSS purchasing and consumption, pre-implementation and post-implementation, found no measurable differences in purchasing or consumption of unhealthy foods and drinks, consumption of alcohol, gambling or payday loans.^21,22^ This study suggests that these null quantitative findings may be due to the ubiquity of adverts for unhealthy commodities from non-LA owned advertising space and displacement of unhealthy advertisements onto these so the overall exposure to advertisements for unhealthy commodities has potentially remained stable, as well as existing advertising restrictions in place for some of the products restricted in this policy (alcohol, payday loans and gambling). To sustain the policy, participants suggested the need for a formal auditing process, continued reviewing and improving of the policy in response to industry adaptations.

### Comparison to existing literature

Our research indicates that some brands adapted their advertising/product portfolio to enable their brand to continue to be advertised, which was an anticipated outcome of this policy in previous work (Scott *et al* 2023).^20^ For example, brands which are strongly associated with HFSS products now advertise their non-HFSS products instead, consistent with the TfL advertising policy evaluation.^18^ Thus, given that outdoor space poses as an ideal advertising space,^26,27^ and when brand advertisements are powerful influencers of product purchasing (rather than product advertisements alone),^28^ this may help explain why we observed limited impact on HFSS purchasing and consumption.

Participants raised that many products, while perceived as HFSS, are classified as non-HFSS by the NPM enabling them to be advertised—indeed products sitting just within the NPM values is something that was observed in our wider quantitative study.^22^ The NPM was developed for food regulatory purposes, however research has shown that it may fail to distinguish between healthy and unhealthy products.^29^ Similarly, issues with the NPM were also identified as barriers for the implementation and impact of the TfL policy.^30^ The authors conclude that adaptations to the NPM are required to both better account for the health offering of food products and be used for outdoor advertising restrictions policies.^30^ Since the evaluation of these policies, the NPM has been updated and now includes more stringent criteria.^31^ However, consistent with the recommendations of the TfL policy evaluation, LAs adopting similar policies would benefit from having a system in place to account for advertisements which are compliant according to the model but not in line with the essence of the policy.^30^ For example, the NPM could be used alongside different models classifying healthy/unhealthy food and drinks products such as WHO NPM or core/non-core to reduce the likelihood of unhealthy foods continuing to be advertised.^32–34^

A key factor which limited the potential effectiveness of the policy was the volume of advertising space owned by the LA. Our interviewees reported that Bristol-City-Council owned approximately one third of outdoor advertising space in the city, leaving a large portion of advertising space unaffected by this policy. On the contrary, when looking at the policy in London which reported a decrease in household HFSS purchasing, the entirety of the TfL estate was within scope.^18,35^ This may suggest that a larger intervention ‘dose’ would be needed for impacts on behaviour change.

### Future research

Our work has identified several avenues for future research. First, it is important to understand the implementation and effectiveness of advertising restriction policies across multiple local authorities with differing governance structures, policy designs and levels of advertising control. Such work would help identify which policy features (eg, scope of coverage, enforcement mechanisms, integration with other policies) are most effective in reducing exposure to unhealthy commodity advertising and influencing behaviour. Second, we need more detailed work to understand how advertising restrictions policies work within a collective ‘whole systems approach’ rather than in isolation (as so in the current study). This includes examining how such policies interact with other interventions (eg, pricing policies, planning regulations, public health campaigns) and whether combined approaches produce effects on behaviour and health outcomes. Third, given the lack of engagement from industry in this research, further work should examine how industry is responding to policies such as these, including strategic adaptation of marketing practices, product reformulation and shifts in advertising placement and messaging.

### Policy implications

Our findings highlight several key considerations for local authorities seeking to develop or strengthen advertising restriction policies, which are summarised in box 1. Our findings emphasise the value of situating responsibility for implementation within appropriate LA teams, maintaining oversight of advertising contracts and addressing organisational barriers such as fragmented ownership and competing priorities. Stakeholders emphasised the importance of formal monitoring and enforcement mechanisms to ensure compliance. Our findings demonstrate a key constraint on policy impact was the limited coverage of LA-owned advertising space, supporting wider evidence that policy ‘dose’ is likely to influence effectiveness, as seen in more comprehensive systems such as the TfL estate; therefore, extending influence through planning regulations, partnership working and coordinated adoption across neighbouring areas may help address displacement. Our findings also highlight the need for iterative policy development in response to adaptive industry practices, reinforcing the importance of regular review and cross-authority learning to identify and close emerging loopholes.

Box 1 **Recommendations for other LAs adopting an advertising restrictions policy**

Recommendations to support implementation

1. Develop a formal auditing process for outdoor advertisements to ensure compliance.
2. Regularly review policy to identify loopholes or industry adaptations which enable the advertising of less healthy products to continue. Embed knowledge sharing with other LAs to do this.
3. Review how the policy can adapt in response to local implementation challenges.
4. Consider adaptation of the processes to distinguish between healthy and unhealthy foods. This is to capture advertisements which, although not classified by the NPM as HFSS, are not in line with the aims of the policy. Or have a decision-making body for such advertisements.
5. Where possible house the policy within the LAs communications team, with support from public health, due to knowledge of advertising contracts and regulations.
6. Where the geographical area of a city encompasses more than one LA, encourage surrounding LAs to implement an advertising restrictions policy to reduce displacement of unhealthy advertisements to these surrounding LAs.
7. Encourage city partners such as universities, leisure centres, bus and train companies to adopt a similar policy to further reduce the displacement of advertisements.
8. Consider the restriction of high-carbon products or activities alongside other unhealthy commodities (eg, energy drinks).
9. Maintain an up-to-date and live list of all advertising and sponsorship contracts, including when they are due to terminate, to ensure timely enactment and enforcement of the policy.

HFSS, high in fat, salt and/or sugar; LA, local authority; NPM, nutrient profile model.

### Strengths and limitations

We interviewed a diverse range of participants from the LA, third-sector, OHID and advertising industry to explore the policy from varied, and often opposing, perspectives. The qualitative study contributes to our mixed-methods natural experimental evaluation, allowing qualitative findings to provide important contextual and explanatory insight into observed quantitative outcomes. At the time of the interviews, participants were unaware of the findings from the quantitative work which mitigates the likelihood that participants’ responses were influenced by knowledge of the policy’s measured effectiveness.

Despite extensive efforts, we were not able to recruit stakeholders working for food/drink manufacturers and representation from the advertising industry was limited and the findings should be interpreted in light of this. Additionally, the participants from the third sector were from organisations who support such policies; there were no representatives from those with other views. The study focuses on a single LA, and findings are therefore shaped by the specific governance structures, policy environment and advertising landscape in Bristol. While this may limit direct transferability to other settings, many of the challenges identified, such as fragmented advertising ownership, industry adaptation and competing institutional priorities, are likely to be relevant to other LAs implementing similar policies.

## Conclusions

Participants reported that this policy increased pressure for further changes to advertising both within and outside of the LA and has contributed to the LA’s whole systems approach to support public health. Industry adaptations to the policy, displacement of advertisements, existing advertising restrictions and limited scope for change on outdoor advertising in the city may have limited the policy’s impact. Such public health policies would benefit from a formal auditing process, adaptations to the process to distinguish between unhealthy and healthy food and drinks, continued reviewing and encouraging surrounding LAs and city partners to adopt a similar policy.

## Supplementary material

10.1136/bmjph-2025-004102 online supplemental file 1

## Data Availability

Data are available upon reasonable request.
