# Supplementary material for: Understanding the implementation, impact and sustainability of Bristol’s unhealthy commodity advertising restrictions policy: a qualitative systems-based study of stakeholder perspectives
Source: BMJ Public Health. 2026 Jun 29;4(2):e004102. doi: 10.1136/bmjph-2025-004102 (PMC13330893; doi:10.1136/bmjph-2025-004102)
Supplement: online supplemental file 1 [file bmjph-4-2-s001.docx]

Supplementary file to: Understanding the implementation, impact, and sustainability of Bristol’s unhealthy commodity advertising restrictions policy: a qualitative systems‑based study of stakeholder perspectives

**Supplementary File 1: Consolidated criteria for reporting qualitative studies (COREQ): 32-item checklist**

**Consolidated criteria for reporting qualitative studies (COREQ): 32-item checklist (1)**

| **No. Item** | **Guide questions/description** | **Response** |
| --- | --- | --- |
| Domain 1: Research team and reﬂexivity |  |  |
| *Personal Characteristics* |  |  |
| 1. Interviewer/facilitator | Which author/s conducted the interview or focus group? | The interviews were conducted by Sarah Harding (SH). |
| 2. Credentials | What were the researcher’s credentials? E.g. PhD, MD | SH, James Nobles (JN), Jeremy Horwood (JH) and Rowan Brockman (RB) form the qualitative research team. All have a PhD. |
| 3. Occupation | What was their occupation at the time of the study? | SH and RB are Senior Research Associates employed at the University of Bristol.  JH is a professor employed at the University of Bristol.  JN is a Senior Research Fellow employed at Leeds Beckett University. |
| 4. Gender | Was the researcher male or female? | SH and RB are female, JN and JH are male. |
| 5. Experience and training | What experience or training did the researcher have? | All researchers in the qualitative research team have undergone training in and teach qualitative research methods. They also have between 8 and 25 years’ experience in qualitative research. |
| *Relationship with participants* |  |  |
| 6. Relationship established | Was a relationship established prior to study commencement? | There was no relationship between the researchers and the participants prior to commencement of the study. The researcher contacted the participants via email to invite them to participate and answered any questions they had prior to the interview. |
| 7. Participant knowledge of the interviewer | What did the participants know about the researcher? e.g. personal goals, reasons for doing the research | Potential participants were invited to participate via email and were provided with a participant information sheet detailing the purpose of the research and the research team and the name of the interviewer. The researcher outlined the reason for doing the research at the start of the interview. |
| 8. Interviewer characteristics | What characteristics were reported about the interviewer/facilitator? e.g. Bias, assumptions, reasons and interests in the research topic | All qualitative researchers were white, British and university educated. It is unlikely that these characteristics add biases to this research. SH kept a reflexivity journal to keep check of any biases. Three researchers were involved in the coding process to add rigour to the analysis process. |
|  |  |  |
| Domain 2: study design |  |  |
| Theoretical framework |  |  |
| 9. Methodological orientation and Theory | What methodological orientation was stated to underpin the study? e.g. grounded theory, discourse analysis, ethnography, phenomenology, content analysis | Thematic analysis was used to analyse the data. |
| Participant selection |  |  |
| 10. Sampling | How were participants selected? e.g. purposive, convenience, consecutive, snowball | Purposive sampling was initially used to recruit stakeholders either directly involved in the policy development/implementation or who were affected by its enactment. Snowball sampling was utilised to identify further stakeholders. |
| 11. Method of approach | How were participants approached? e.g. face-to-face, telephone, mail, email | Potential participants were invited to participate via email and were provided with a participant information sheet detailing the purpose of the research, the research team and what participation involved. |
| 12. Sample size | How many participants were in the study? | N=22 |
| 13. Non-participation | How many people refused to participate or dropped out? Reasons? | An additional 2 employees from third-sector and 1 from Bristol-City-Council were approached and declined an interview and 23 food and/or drinks companies and 1 other advertising company were approached and either declined an interview (n=2) or did not respond (n=22). |
| Setting |  |  |
| 14. Setting of data collection | Where was the data collected? e.g. home, clinic, workplace | All interviews were carried out online by SH (using MS Teams or Zoom), |
| 15. Presence of non-participants | Was anyone else present besides the participants and researchers? | No |
| 16. Description of sample | What are the important characteristics of the sample? e.g. demographic data, date | See Table 1 in the main paper. |
| Data collection |  |  |
| 17. Interview guide | Were questions, prompts, guides provided by the authors? Was it pilot tested? | The interview topic guide was developed within the research team and through stakeholder discussion. Specific interview questions were tailored to each participant’s expertise and knowledge. |
| 18. Repeat interviews | Were repeat interviews carried out? If yes, how many? | No |
| 19. Audio/visual recording | Did the research use audio or visual recording to collect the data? | All interviews were audio-recorded. |
| 20. Field notes | Were ﬁeld notes made during and/or after the interview or focus group? | Throughout the data collection and analysis process, SH kept a record of reflexive notes, impressions of the data, and thoughts about the data collection and analysis. |
| 21. Duration | What was the duration of the interviews or focus group? | Interviews ranged from 25-60 minutes |
| 22. Data saturation | Was data saturation discussed? | Data were collected until the data saturation was reached and this was determined by discussions among the research team involved in data analysis (SH, JN, and RB) |
| 23. Transcripts returned | Were transcripts returned to participants for comment and/or correction? | In response to a request, one participant viewed their interview transcript for clarification and corrections. |
| Domain 3: analysis and ﬁndings |  |  |
| Data analysis |  |  |
| 24. Number of data coders | How many data coders coded the data? | There were 3 data coders (SH, JN and RB) |
| 25. Description of the coding tree | Did authors provide a description of the coding tree? | Initial codes and coding framework were developed inductively by the lead researcher (SH) and four interview transcripts were second coded by JN and RB to refine the coding framework. The themes were generated based on the codes. The major and minor themes are described in the main paper. |
| 26. Derivation of themes | Were themes identiﬁed in advance or derived from the data? | Themes were developed inductively from the data. |
| 27. Software | What software, if applicable, was used to manage the data? | We used NVivo (version 14) to support data analysis. |
| 28. Participant checking | Did participants provide feedback on the ﬁndings? | No. |
| Reporting |  |  |
| 29. Quotations presented | Were participant quotations presented to illustrate the themes/ﬁndings? Was each quotation identiﬁed? e.g. participant number | Yes. Each theme is supported by an illustrative quote in the main paper. |
| 30. Data and ﬁndings consistent | Was there consistency between the data presented and the ﬁndings? | Yes. The findings in the main paper are supported by data (quotations). |
| 31. Clarity of major themes | Were major themes clearly presented in the ﬁndings? | Yes, they are presented in the main paper. |
| 32. Clarity of minor themes | Is there a description of diverse cases or discussion of minor themes? | Yes, they are presented in the main paper. |

1. Tong A, Sainsbury P, Craig J. Consolidated criteria for reporting qualitative research (COREQ): a 32-item checklist for interviews and focus groups. International journal for quality in health care. 2007;19(6):349-57.
